# ASGAL: aligning RNA-Seq data to a splicing graph to detect novel alternative splicing events

**DOI:** 10.1186/s12859-018-2436-3

**Published:** 2018-11-20

**Authors:** Luca Denti, Raffaella Rizzi, Stefano Beretta, Gianluca Della Vedova, Marco Previtali, Paola Bonizzoni

**Affiliations:** 1 Department of Informatics, Systems, and Communication, University of Milano - Bicocca, Milan, Italy; 2 Institute for Biomedical Technologies, National Council of Research, Segrate, Italy

**Keywords:** Graph alignment, Spliced alignment, Alternative splicing events, RNA-Seq

## Abstract

**Background:**

While the reconstruction of transcripts from a sample of RNA-Seq data is a computationally expensive and complicated task, the detection of splicing events from RNA-Seq data and a gene annotation is computationally feasible. This latter task, which is adequate for many transcriptome analyses, is usually achieved by aligning the reads to a reference genome, followed by comparing the alignments with a gene annotation, often implicitly represented by a graph: the *splicing graph*.

**Results:**

We present ASGAL (Alternative Splicing Graph ALigner): a tool for mapping RNA-Seq data to the splicing graph, with the specific goal of detecting novel splicing events, involving either annotated or unannotated splice sites. ASGAL takes as input the annotated transcripts of a gene and a RNA-Seq sample, and computes (1) the spliced alignments of each read in input, and (2) a list of novel events with respect to the gene annotation.

**Conclusions:**

An experimental analysis shows that ASGAL allows to enrich the annotation with novel alternative splicing events even when genes in an experiment express at most one isoform. Compared with other tools which use the spliced alignment of reads against a reference genome for differential analysis, ASGAL better predicts events that use splice sites which are novel with respect to a splicing graph, showing a higher accuracy. To the best of our knowledge, ASGAL is the first tool that detects novel alternative splicing events by directly aligning reads to a splicing graph.

**Availability:**

Source code, documentation, and data are available for download at http://asgal.algolab.eu.

## Background

Data coming from high-throughput sequencing of RNA (RNA-Seq) can shed light on the diversity of transcripts that results from Alternative Splicing (AS). Computational approaches for transcriptome analysis from RNA-Seq data may be classified according to two primary goals: (i) detection of AS events and (ii) full-length isoform reconstruction. Tools in these two categories may be further classified based on an approach which may be (a) de-novo assembly based or (b) gene annotation guided or reference based. Various tools have been proposed in the literature that fall in the categories listed above. Examples of tools in category (ii.a) that do not require a reference genome are Trinity [1] and ABySS [2], while Cufflinks [3], Scripture [4], and Traph [5], among many others, are known tools of category (ii.b). The first two tools were originally designed for de-novo isoform prediction and can make limited use of existing annotations. While the reconstruction of full-length transcripts (either de-novo or using a reference) is a computationally intensive task, the detection of AS events is computationally feasible and it can be achieved without performing intensive steps related to transcript reconstruction. Observe that given a set of transcripts reconstructed from a sample of RNA-Seq reads, a tool for comparing transcripts is needed to extract AS events. Such a comparison is performed for example by AStalavista [6], a popular tool for the exhaustive extraction and visualization of complex AS events from full-length transcripts. This tool does not use RNA-Seq reads as input but only the gene annotation, and it does not focus on single events (such as exon skipping, alternative splice sites, etc.) but rather uses a flexible coding of AS events [7] to list all the AS events between each pair of transcripts.

Since reconstructing full-length isoforms from RNA-Seq reads is a difficult and computationally expensive problem, one may restrict the task to the direct detection of AS events from RNA-Seq data through an alignment process. Following the latter approach, we propose a computational approach to predict AS events, and we implement this procedure in a tool — ASGAL — belonging to category (i.b). Compared to existing tools, ASGAL has as main goals the splice-aware alignment of RNA-Seq data to a splicing graph and the annotation of the graph with novel splicing events that are supported by such alignments. From this perspective, differently from tools for event detection based on differential analysis, ASGAL is able to detect a novel event in a gene annotation when this event is supported by reads from a single unannotated isoform. Some tools using unannotated splice sites — hence most similar to ASGAL with respect to the goal of predicting AS events — are SpliceGrapher [8] and SplAdder [9] which take as input the spliced alignments of sequencing data (RNA-Seq data for SplAdder, and RNA-Seq data in addition to EST data for SpliceGrapher) against a reference genome, and produce an augmented graph representation of the annotated transcripts, traditionally known as the *splicing graph* [10], with nodes and edges that may represent novel AS events. The main task of SplAdder is the prediction of AS events that are supported by an input sample, and the quantification of those events by testing the differences between multiple samples. Two other tools whose main goal is differential alternative splicing analysis are SUPPA2 [11] and rMATS [12]. Both SUPPA2 and rMATS analyze RNA-Seq data from different samples (replicates) to obtain the set of differential alternative splicing events between the analyzed conditions. SUPPA2 is only able to detect AS events that are in the annotation, while rMATS only lists novel events that use annotated splice sites. Similarly, MAJIQ [13] analyzes RNA-Seq data and a set of (annotated) transcripts to quantify the relative abundances of a set of Local Splicing Variations which implicitly represent combinations of AS events involving both annotated and novel splice sites, but also changes of these abundances between conditions. Note that both MAJIQ and rMATS do not include an alignment step, but need an external spliced aligner such as STAR [14], while SUPPA2 requires the quantification of the input transcripts, which can be obtained by using a tool like Salmon [15]. In both cases, the identification of AS events stems from an analysis of the expression levels. A most recent tool, LeafCutter [16] analyzes RNA-Seq data and quantifies differential intron usage across samples, allowing the detection of novel introns which model complex splicing events. Like the other cited tools, LeafCutter requires as input the spliced alignments of the RNA-Seq samples of interest. Two crucial computational instruments are usually required by tools of category (i.b): an input file consisting of the alignment of RNA-Seq data to a reference genome, and a gene annotation. The first input may significantly change the performance of such tools, as the accuracy of the alignment may affect the predictions of AS events. In particular, the alignment to a reference genome is usually guided by the annotated transcripts that may be represented by a splicing graph that is then enriched with the information coming from the computed alignments.

With the main goal of enriching a gene annotation with novel AS events supported by a RNA-Seq sample, we investigated an alternative approach that directly aligns the input reads against a splicing graph representing a gene annotation. The main motivation of our proposal is that, by using the splicing graph during the alignment phase, we are able to obtain an alignment focused on enriching a gene annotation with AS events that produce novel isoforms by using annotated or unannotated splice sites with respect to the actual graph. For this purpose, we implemented ASGAL (Alternative Splicing Graph ALigner), a tool that consists of two parts: (i) a splice-aware aligner of RNA-Seq reads to a splicing graph, and (ii) a predictor of AS events supported by the RNA-Seq mappings. Currently, there are several tools for the spliced alignment of RNA-Seq reads against a reference genome or a collection of transcripts but, to the best of our knowledge, ASGAL is the first tool specifically designed for mapping RNA-Seq data directly to a splicing graph. Differently from SplAdder, which enriches a splicing graph representing the gene annotation using the splicing information contained in the input spliced alignments, and then analyzes this enriched graph to detect the AS events differentially expressed in the input samples, ASGAL directly aligns the input sample to the splicing graph of the gene of interest and then detects the AS events which are novel with respect to the input gene annotation, comparing the obtained alignments with it. More precisely, ASGAL extracts the introns supported by the alignments of reads against the splicing graph, then compares them against the input annotation to detect whether novel events may be predicted from the input reads. This allows ASGAL to detect novel event types even when the input RNA-Seq sample consists only of reads that are not consistent with the input splicing graph, because of the AS event, provided that the number of alignments confirming the AS event is above a certain threshold. Instead, SplAdder needs to receive reads originating from both transcripts that induce the AS event.

The approach of inferring AS events directly from RNA-Seq reads, without assembling isoforms, is also proposed in [17], where the main idea is to perform a de-novo prediction of some AS events from the De Brujin graph assembly of RNA-Seq data, i.e. without using any gene annotation. An investigation of the de-novo prediction of AS events directly from RNA-Seq data is also given in [18], where a characterization of the splicing graph that may be detected in absence of a gene annotation (either given as a reference or as a list of transcripts) is provided.

The ASGAL mapping algorithm improves a previous solution to the *approximate pattern matching to a hypertext* problem (an open problem faced in [19]). The approximate matching of a string to a graph with labeled vertices is a computational problem first introduced by Manber and Wu [20] and attacked by many researchers [21–23]. Navarro [24] improved all previous results in both time and space complexity, proposing an algorithm which requires $\mathcal {O}{m(n+e)}$ time, where *m* is the length of the pattern, *n* is the length of the concatenation of all vertex labels, and *e* is the total number of edges. The method in [19] improves the latest result by Thachuk [25]: an algorithm with time complexity $\mathcal {O}{m + \gamma ^{2}}$ using succinct data structures to solve the exact version of matching a pattern to a graph — i.e. without errors — where *γ* is the number of occurrences of the node texts as substrings of the pattern. The algorithm in [19] is based on the concept of *Maximal Exact Match* and it uses a succinct data structure to solve the approximate matching of a pattern to a hypertext in $\mathcal {O}{m+\eta ^{2}}$ time, where *η* is the number of Maximal Exact Matches between the pattern and the concatenation of all vertex labels. In this paper, we improve the results in [19] by extending the algorithm to implement a RNA-Seq data aligner for detecting general AS event types from the splicing graph.

An experimental analysis on real and simulated data was performed with the purpose of assessing the quality of ASGAL in detecting AS event types that are annotated or novel with respect to a gene annotation. We note that the current implementation of ASGAL is not able to detect the insertion of novel exons inside an intron and intron retention events caused by the union of two exons. In the first part of our experimental analysis, we compared the alignment step of ASGAL with STAR, one of the best-known spliced aligner. The results show a good accuracy of ASGAL in producing correct alignments by directly mapping the RNA-Seq reads against the splicing graph of a gene. Although ASGAL works under different assumptions than other existing tools, we decided to compare ASGAL with SplAdder, rMATS, and SUPPA2. For this purpose we first ran an experimental analysis on simulated data and compared the event identification step of ASGAL. We performed two distinct analysis. In the first one, we evaluated the accuracy of the tools in predicting novel AS event types, i.e. events that are not already contained in the annotation. Instead in the second analysis, all the tools were compared to assess their accuracy in detecting AS events that are already present in the input annotation and are supported by the RNA-Seq experiments. We also ran an experimental analysis on real data with the main goal of evaluating ASGAL, SplAdder, rMATS, and SUPPA2 in identifying RT-PCR validated alternative splicing events. We performed this last experiment also to test the ability of ASGAL in detecting such events as novel ones, that is by removing the events from the input annotation and keeping their evidence only in the RNA-Seq data.

The results in the simulated scenario show that ASGAL achieved the best values of precision, recall and F-measure in predicting alternative splicing events supported by the reads that are novel compared to the annotation specified by a splicing graph. The results on real data show the ability of ASGAL to detect RT-PCR validated alternative splicing events when they are simulated as novel events with respect to the annotated splicing graph.

## Methods

ASGAL (Alternative Splicing Graph ALigner) is a tool for performing a mapping of RNA-Seq data in a sample against the splicing graph of a gene with the main goal of detecting novel alternative splicing events supported by the reads of the sample with respect to the annotation of the gene. More precisely, ASGAL takes as input the annotation of a gene together with the related reference sequence, and a set of RNA-Seq reads, to output (i) the spliced alignments of each read in the sample and (ii) the alternative splicing events supported by the sample which are novel with respect to the annotation. We point out that ASGAL uses the input reference sequence only for building the splicing graph as well as for refining the alignments computed against it, with the specific goal of improving the precision in the AS event type detection. Each identified event is described by its type, i.e. exon skipping, intron retention, alternative acceptor splice site, alternative donor splice site, its genomic location, and a measure of its quantification, i.e. the number of alignments that support the identified event.

This section is organized as follows. We first introduce the basic definitions and notions that we will use in the section *spliced graph-alignment*, and finally we describe the steps of our method. For the sake of clarity, we will describe our method considering as input the splicing graph of a single gene: it can be easily generalized to manage more than a gene at a time. However, the current version of ASGAL tool cannot manage more than a limited set of genes. At the end of this section, we will propose a possible procedure an user can adopt to use our tool in a genome-wide analysis.

### Definitions

From a computational point of view, a genome is a sequence of characters, i.e. a string, drawn from an alphabet of size 4 (A, C, G, and T). A gene is a *locus* of the genome, that is, a gene is a substring of the genome. Exons and introns of a gene locus will be uniquely identified by their starting and ending positions on the genome. A transcript *T* of gene *G* is a sequence 〈[*a*_1_,*b*_1_],[*a*_2_,*b*_2_],…,[*a*_*n*_,*b*_*n*_]〉 of exons on the genome, where *a*_*i*_ and *b*_*i*_ are respectively the *start* and the *end* positions of the *i*-th exon of the transcript. Observe that *a*_1_ and *b*_*n*_ are the *starting* and *ending positions* of transcript *T* on the genome, and each [*b*_*i*_+1,*a*_*i*+1_−1] is an *intron* represented as a pair of positions on the genome. In the following, we denote by $\mathcal {E}_{G}$ the set of all the exons of the transcripts of gene *G*, that is $\mathcal {E}_{G}=\cup _{T \in \mathcal {T}}\mathcal {E}(T)$, where $\mathcal {E}(T)$ is the set of exons of transcript *T* and $\mathcal {T}$ is the set of transcripts of *G*, called the *annotation* of *G*. Given two exons *e*_*i*_=[ *a*_*i*_,*b*_*i*_] and *e*_*j*_=[ *a*_*j*_,*b*_*j*_] of $\mathcal {E}_{G}$, we say that *e*_*i*_*precedes* *e*_*j*_ if *b*_*i*_<*a*_*j*_ and we denote this by *e*_*i*_≺*e*_*j*_. Moreover, we say that *e*_*i*_ and *e*_*j*_ are *consecutive* if there exists a transcript $T \in \mathcal T$ and an index *k* such that *e*_*k*_=*e*_*i*_ and *e*_*k*+1_=*e*_*j*_, and *e*_*i*_, *e*_*j*_ in $\mathcal {E}(T)$.

**Fig. 1:**
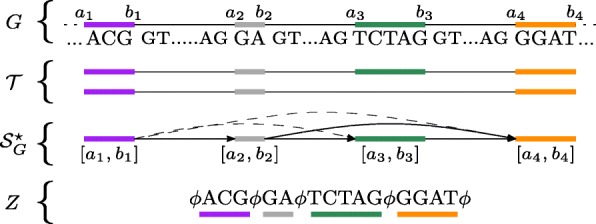
Example of Splicing Graph. A simple gene *G* with 4 exons is shown along with its annotation (transcripts) $\mathcal {T}$, the corresponding splicing graph $\mathcal {S}_{G}^{\star }$, and the linearization *Z*. In $\mathcal {S}_{G}^{\star }$, dashed arrows represent the novel edges while full arrows represent the edges contained in $\mathcal {S}_{G}$

The *splicing graph* of a gene *G* is the directed acyclic graph $\mathcal {S}_{G} = (\mathcal {E}_{G},E)$, i.e. the vertex set is the set of the exons of *G*, and the edge set *E* is the set of pairs (*v*_*i*_,*v*_*j*_) such that *v*_*i*_ and *v*_*j*_ are consecutive in at least one transcript. For each vertex *v*, we denote by seq(*v*), the genomic sequence of the exon associated to *v*. Finally, we say that $\mathcal {S}_{G}^{\star }$ is the graph obtained by adding to $\mathcal {S}_{G}$ all the edges (*v*_*i*_,*v*_*j*_)∉*E* such that *v*_*i*_≺*v*_*j*_. We call these edges *novel* edges. Note that the novel edges represent putative novel junctions between two existing exons (that are not consecutive in any transcript of *G*). Figure 1 shows an example of the definitions of gene, exon, annotation, and splicing graph.

**Fig. 2:**
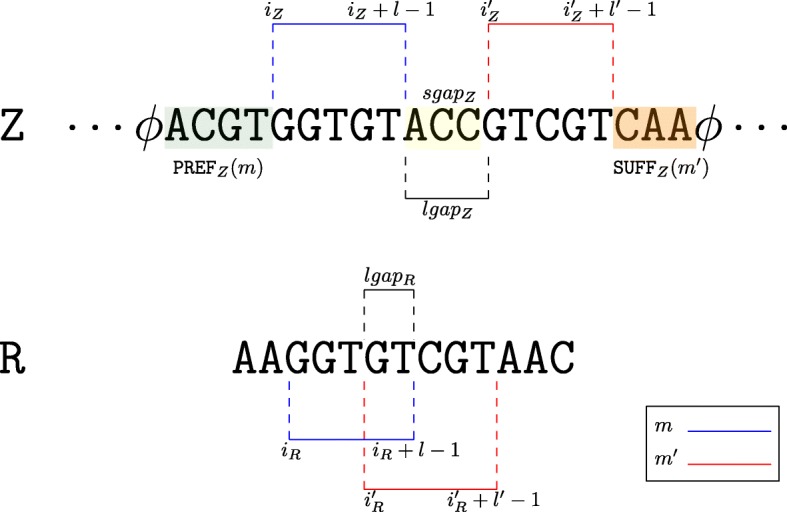
Precedence relation between MEMs. Two MEMs, *m*=(*i*_*Z*_,*i*_*R*_,*l*) and $m^{\prime } = \left (i^{\prime }_{Z}, i^{\prime }_{R}, l^{\prime }\right)$, are shown in the figure. For ease of presentation we represent in blue the former and in red the latter. Since $i_{Z} < i^{\prime }_{Z}$ and $i_{Z} + l < i^{\prime }_{Z} + l^{\prime }$, *m* precedes *m*^′^ on Z; analogously *m* precedes *m*^′^ on *R* since $i_{R} < i^{\prime }_{R}$ and $i_{R} + l < i^{\prime }_{R} + l^{\prime }$. The length *lgap*_*Z*_ of the gap between the two MEMs on *Z* is positive and we refer to the string between *i*_*Z*_+*l* and $i^{\prime }_{Z} - 1$ as *sgap*_*Z*_ (highlighted in yellow). Conversely, the length of the gap between the two MEMs on *R* is negative and we say that they overlap on *R*. Finally, we refer to the string between the start of the vertex label and *i*_*Z*_−1 as PREF_*Z*_(*m*) (highlighted in light blue), and to the string between $i^{\prime }_{Z} + l$ and the end of the vertex label as SUF*F*_*Z*_(*m*^′^) (highlighted in light red). For ease of presentation, we did not report SUFF_*Z*_(*m*) and PREF_*Z*_(*m*^′^)

In the following, we will use the notion of *Maximal Exact Match* (MEM) to perform the spliced graph-alignment of a RNA-Seq read to $\mathcal {S}_{G}$. Given two strings *R* and *Z*, a MEM is a triple *m*=(*i*_*Z*_,*i*_*R*_,*ℓ*) representing the common substring of length *ℓ* between the two strings that starts at position *i*_*Z*_ in *Z*, at position *i*_*R*_ in *R*, and that cannot be extended in either direction without introducing a mismatch. Computing the MEMs between a string *R* and a splicing graph $\mathcal {S}_{G}$ can be done by concatenating the labels of all the vertices and placing the special symbol *ϕ* before each label and after the last one, obtaining a string $Z = \phi {\texttt {seq}}(v_{1}) \phi {\texttt {seq}}(v_{2}) \phi \ldots \phi {\texttt {seq}}(v_{|\mathcal {E}_{G}|}) \phi $ that we call the *linearization* of the splicing graph (see Fig. 1 for an example). It is immediate to see that, given a vertex *v* of $\mathcal {S}_{G}$, the label seq(*v*) is a particular substring of the linearization *Z*. For the sake of clarity, let us denote this substring, which is the one related to seq(*v*), as *Z*[ *i*_*v*_,*j*_*v*_]. Then, by employing the algorithm by Ohlebusch et al. [26], all the MEMs longer than a constant *L* between *R* and *Z*, thus between *R* and $\mathcal {S}_{G}$, can be computed in linear time with respect to the length of the reads and the number of MEMs. Thanks to the special character *ϕ* which occurs in *Z* and not in *R*, each MEM occurs inside a single vertex label and cannot span two different labels. In the following, given a read *R* and the linearization *Z* of $\mathcal {S}_{G}$, we say that a MEM *m*=(*i*_*Z*_,*i*_*R*_,*l*) belongs to vertex *v* if *i*_*v*_≤*i*_*Z*_≤*j*_*v*_ where [ *i*_*v*_,*j*_*v*_] is the interval on *Z* related to the vertex label seq(*v*) (that is, seq(*v*)=*Z*[ *i*_*v*_,*j*_*v*_]). We say that a MEM *m*=(*i*_*Z*_,*i*_*R*_,*l*) precedes another MEM $m^{\prime }=\left (i^{\prime }_{Z}, i^{\prime }_{R}, l^{\prime }\right)$ in *R* if $i_{R} < i^{\prime }_{R}$ and $i_{R} + l < i^{\prime }_{R} + l^{\prime }$, and we denote this by *m*≺_*R*_*m*^′^. Similarly, when *m* precedes *m*^′^ in *Z*, we denote it by *m*≺_*Z*_*m*^′^, if the previous properties hold on *Z* and the two MEMs belong to the same vertex label seq(*v*). When *m* precedes *m*^′^ in *R* (in *Z*, respectively), we say that $lgap_{R} = i^{\prime }_{R} - (i_{R} + l)$ (${lgap}_{Z} = i^{\prime }_{Z} - (i_{Z} + l)$, respectively) is the length of the gap between the two MEMs. If *lgap*_*R*_ or *lgap*_*Z*_ (or both) are positive, we refer to the gap strings as *sgap*_*R*_ and *sgap*_*Z*_, while when they are negative, we say that *m* and *m*^′^*overlap* either in *R* or *Z* (or both). Given a MEM *m* belonging to the vertex labeled seq(*v*), we denote as PREF_*Z*_(*m*) and SUFF_*Z*_(*m*) the prefix and the suffix of seq(*v*) upstream and downstream from the start and the end of *m*, respectively. Figure 2 summarizes the definitions of precedence between MEMs, gap, overlap, PREF_*Z*_, and SUFF_*Z*_.

### Spliced graph-alignment

We are now able to define the fundamental concepts that will be used in our method. In particular, we first define a general notion of gap graph-alignment and then we introduce specific constraints on the use of gaps to formalize a splice-aware graph-alignment that is fundamental for the detection of alternative splicing events in ASGAL.

A *gap graph-alignment* of *R* to graph $\mathcal {S}_{G}$ is a pair (*A*,*π*) where *π*=〈*v*_1_,…,*v*_*k*_〉 is a path of the graph $\mathcal {S}_{G}^{\star }$ and

$$A = \left\langle (p_{1}, r_{1}), \left(p_{1}^{\prime}, r_{1}^{\prime}\right), \ldots, \left(p^{\prime}_{n-1}, r^{\prime}_{n-1}\right),(p_{n}, r_{n}) \right\rangle $$

is a sequence of pairs of strings, with *n*≥*k*, such that seq(*v*_1_)=*x*·*p*_1_ and seq(*v*_*k*_)=*p*_*n*_·*y*, for *x*,*y* possibly empty strings and $P= p_{1} \cdot p_{1}^{\prime } \cdot p_{2} \cdot p_{2}^{\prime } \cdot p_{3} \cdots p_{n-1}^{\prime } \cdot p_{n} $ is the string labeling the path *π* and $R = r_{1} \cdot r_{1}^{\prime } \cdot r_{2} \cdots r_{n-1}^{\prime } \cdot r_{n} $.

The pair (*p*_*i*_,*r*_*i*_), called a *factor* of the alignment *A*, consists of a non-empty substring *r*_*i*_ of *R* and a non-empty substring *p*_*i*_ of the label of a vertex in *π*. On the other hand, the pair $\left (p_{i}^{\prime }, r_{i}^{\prime }\right)$ is called a *gap-factor* of the alignment *A* if at least one of $p_{i}^{\prime }$ and $r_{i}^{\prime }$ is an empty substring *ε*. Moreover, either $p_{i}^{\prime }$ is empty or $|p_{i}^{\prime }| > \alpha $, and either $r_{i}^{\prime }$ is empty or $|r_{i}^{\prime }| > \alpha $, for a fixed value *α* which represents the maximum *alignment indel* size allowed. When an insertion (or a deletion) is smaller than *α*, we consider it an alignment indel and we incorporate it into a factor; otherwise, we consider it as a clue of the possible presence of an AS event and we represent it as a gap-factor. We note that an “alignment indel” is a small insertion or deletion which occurs in the alignment, due to a sequencing error in the input data or a genomic insertion/deletion. Intuitively, in a gap graph-alignment, factors correspond to portions of exons covered (possibly with errors) by portions of the read, while gap-factors correspond to introns, which can be already annotated or novel, and which can be used to infer the possible presence of AS events. We note that to allow the detection of alternative splice site events known as NAGNAG resulting in a difference of 3bps, if an alignment indel occurs at the beginning or at the end of an exon, we consider it during the detection of the events, even though it is not modeled as a gap-factor since in these cases the insertion may be smaller than *α*.

We associate to each factor (*p*_*i*_,*r*_*i*_) the cost *δ*(*p*_*i*_,*r*_*i*_), and to each gap-factor $\left (p_{i}^{\prime },r_{i}^{\prime }\right)$ the cost $\delta \left (p_{i}^{\prime },r_{i}^{\prime }\right)$, by using a function *δ*(·,·) with positive values. Then the cost of the alignment (*A*,*π*) is given by the expression:

$$ {\mathtt{cost}}(A, \pi) = \sum_{i=1}^{n} \delta(p_{i}, r_{i}) + \sum_{i=1}^{n-1} \delta\left(p_{i}^{\prime},r_{i}^{\prime}\right). $$

Moreover, we define the *error* of a gap graph-alignment as the sum of the edit distance of each factor (but not of gap-factors). Formally, the error of the alignment (*A*,*π*) is:

$$ {\mathtt{Err}}(A, \pi) = \sum_{i=1}^{n} d(p_{i}, r_{i}), $$

where *d*(·,·) is the edit distance between two strings.

To define a splice-aware alignment, that we call *spliced graph-alignment*, we need to classify each gap-factor and to assign it a cost. Our primary goal is to compute a gap graph-alignment of the read to the splicing graph that possibly reconciles to the gene annotation; if this is not possible, then we want to minimize the number of novel events. For this reason we distinguish three types of gap-factors: *annotated*, *novel*, and *uninformative*. Intuitively, an annotated gap-factor models an annotated intron, a novel gap-factor represents a novel intron, while an uninformative gap-factor does not represent any intron.

Formally, we classify a gap-factor $\left (p_{i}^{\prime }, r_{i}^{\prime }\right)$ as annotated if and only if $p^{\prime }_{i} = r^{\prime }_{i} = \epsilon $ and the two strings *p*_*i*_, *p*_*i*+1_ are on two different vertices that are linked by an edge in $\mathcal {S}_{G}$. We classify a gap-factor $\left (p_{i}^{\prime }, r_{i}^{\prime }\right)$ as novel in the following cases:

1. $r_{i}^{\prime } = \epsilon $ and $p_{i}^{\prime }= \epsilon $ occurs between the strings *p*_*i*_ and *p*_*i*+1_ which belong to two distinct vertices linked by an edge in $\mathcal {S}_{G}^{\star }$ and not in $\mathcal {S}_{G}$ (i.e. this gap-factor represents an exon skipping event — Fig. 3a).

**Fig. 3:**
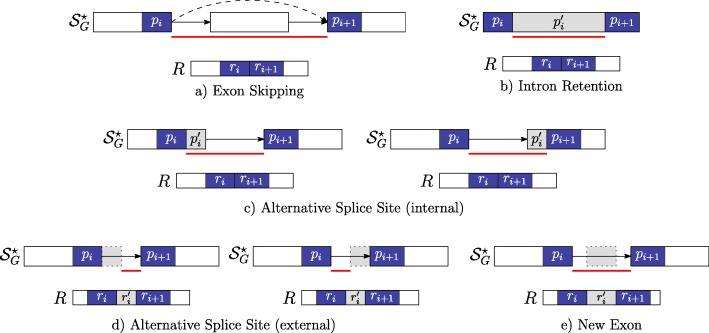
Novel gap-factors. The relationship among novel gap-factors, introns, and AS events is shown. Each subfigure depicts an example of novel gap-factor $\left (p^{\prime }_{i},r^{\prime }_{i}\right)$ (gray boxes) in relation to a simple graph $\mathcal {S}_{G}^{\star }$, where dashed arrows represent novel edges (not present in the splicing graph $\mathcal {S}_{G}$) and a read *R*. The two consecutive factors (*p*_*i*_,*r*_*i*_) and (*p*_*i*+1_,*r*_*i*+1_) of a spliced graph-alignment are represented by blue boxes, and the red lines represent the novel introns supported by the gap-factors. In terms of novel AS events, gap-factor (*ε*,*ε*) in case **a** supports an exon skipping, gap-factor $\left (p^{\prime }_{i}, \epsilon \right)$ supports an intron retention in case **b** and alternative splice sites shortening an exon in case **c**. Finally, gap-factor $\left (\epsilon, r^{\prime }_{i}\right)$ supports alternative splice sites extending an exon in case **d** and a new exon in case **e**
2. $r_{i}^{\prime } = \epsilon $ and $p_{i}^{\prime }\neq \epsilon $ occurs between the strings *p*_*i*_ and *p*_*i*+1_ which belong to the same vertex of $\mathcal {S}_{G}^{\star }$ (i.e. this gap-factor represents an intron retention event — Fig. 3b). Actually, we note here that this type of gap-factor may represent also a genomic deletion: currently, our program does not distinguish between intron retentions and genomic deletions that are entirely contained in an exon, therefore we might overpredict intron retentions.
3. $r_{i}^{\prime } = \epsilon $ and $p_{i}^{\prime }\neq \epsilon $ occurs between the strings *p*_*i*_ and *p*_*i*+1_ which belong to two distinct vertices linked by an edge in $\mathcal {S}_{G}^{\star }$ (i.e. this gap-factor represents an alternative splice site event — Fig. 3c).
4. $r_{i}^{\prime } \ne \epsilon $ and $p_{i}^{\prime }= \epsilon $ occurs between the strings *p*_*i*_ and *p*_*i*+1_ which belong to two distinct vertices linked by an edge in $\mathcal {S}_{G}^{\star }$ (i.e. this gap-factor represents an alternative splice site extending an exon or a new exon event — Fig. 3d-e).

Note that Case 1 allows to detect a novel intron whose splice sites are both annotated (see Fig. 3a). Case 2 supports a genomic deletion or an intron retention (see Fig. 3b), and in case of intron retention, ASGAL finds the two novel splice sites inside the annotated exon. Case 3 gives an evidence of a novel alternative splice event shortening an annotated exon (see Fig. 3c) and ASGAL finds the novel splice site supported by this case. Finally, in Case 4, ASGAL is able to detect a novel alternative splice site (extending an annotated exon) or a novel exon (see Fig. 3d), but only in the first case (alternative splice site) ASGAL is able to find the novel splice site induced by the gap-factor.

**Fig. 4:**
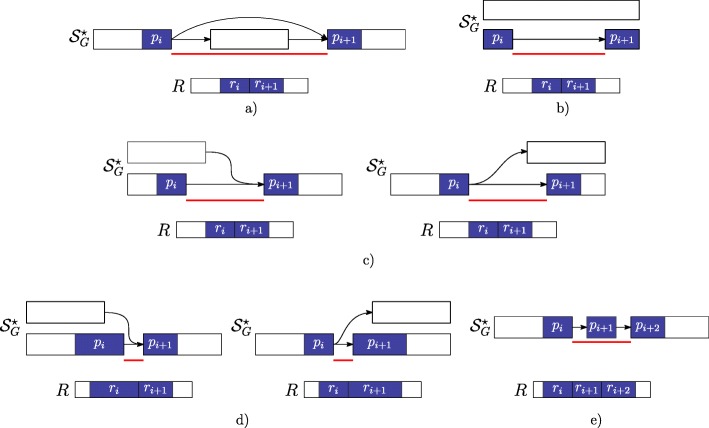
Annotated gap-factors. The novel gap-factors of Fig. 3 are shown in their annotated counterpart. Observe that now they are all (*ε*,*ε*) and are annotated as well as the supported introns (red lines) and the related AS events. **a** Exon Skipping **b** Intron Retention **c** Alternative Splice Site (internal) **d** Alternative Splice Site (external) **e** New Exon

For ease of presentation, Fig. 3 shows only “classic” AS event types and not their combination as those modeled with the notion of Local Splicing Variations (LSV) [13]. We note here that our formalization takes into account combinations of AS event types as those given by an exon skipping combined with an alternative splice site (see definition of gap-factor in cases 3 and 4). However, the actual version of the tool is designed only to detect the AS event types shown in Fig. 3. For completeness, in Fig. 4 we show the same AS event types (shown in Fig. 3) with respect to the annotated case, i.e. when the gap-factor is annotated and it represents an already known AS event.

Finally, we classify a gap-factor $\left (p^{\prime }_{i}, r^{\prime }_{i}\right)$ as uninformative in the two remaining cases, which are (i) $r_{i}^{\prime } = \epsilon $ and $p_{i}^{\prime }= \epsilon $ occurs between strings *p*_*i*_ and *p*_*i*+1_ which belong to the same vertex, and (ii) $r_{i}^{\prime } \neq \epsilon $ and $p_{i}^{\prime }= \epsilon $ occurs between strings *p*_*i*_ and *p*_*i*+1_ which belong to the same vertex. We notice that in the former case, factors (*p*_*i*_,*r*_*i*_) and (*p*_*i*+1_,*r*_*i*+1_) can be joined into a unique factor.

**Fig. 5:**
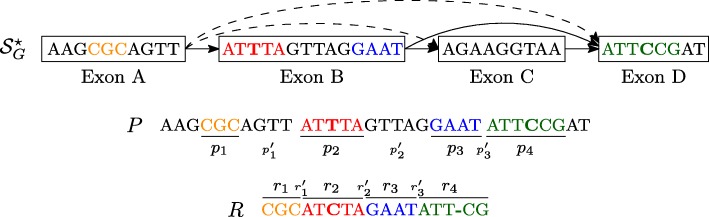
Spliced graph-alignment. Example of a spliced graph-alignment of a read *R* to a splicing graph $\mathcal {S}_{G}^{\star }$ (in $\mathcal {S}_{G}^{\star }$, the dashed arrows represent the novel edges not present in $\mathcal {S}_{G}$). The read *R* has been factorized in four strings *r*_1_, *r*_2_, *r*_3_, and *r*_4_ matching to strings *p*_1_, *p*_2_, *p*_3_, and *p*_4_ of *P*, which is the concatenation of exon labels of path *π*=〈*A*,*B*,*D*〉. This yields to the spliced graph-alignment $\left (\left \langle (p_{1},r_{1}), \left (p_{1}^{\prime }, r_{1}^{\prime }\right), (p_{2},r_{2})\right.\right.$, $\left.\left.\left (p_{2}^{\prime }, r_{2}^{\prime }\right), (p_{3},r_{3}), \left (p_{3}^{\prime }, r_{3}^{\prime }\right), (p_{4},r_{4})\right \rangle, \pi \right)$. We observe that $p^{\prime }_{3}$, $r^{\prime }_{1}$, $r^{\prime }_{2}$, and $r^{\prime }_{3}$ are equal to *ε*. Moreover, we note that $\left (p_{1}^{\prime }, r_{1}^{\prime }\right)$, $\left (p_{2}^{\prime },r_{2}^{\prime }\right)$ are two novel gap-factors, *r*_2_ matches *p*_2_ with an error of substitution while *r*_4_ matches *p*_4_ with an error of insertion: both the error and the cost of this spliced-graph alignment are equal to 2. This alignment of *R* to the splicing graph of *G* supports the evidence of two *novel* alternative splicing events: an alternative donor site of exon *A* and an intron retention on exon *B*

Let $\mathcal {G_{F}}$ be the set of novel gap-factors of a gap graph-alignment *A*. Then a *spliced graph-alignment* (*A*,*π*) of *R* to $\mathcal {S}_{G}$ is a gap graph-alignment in which uninformative gap-factors are not allowed, whose cost is defined as the number of novel gap-factors, and whose error is at most *β*, for a given constant *β* which models any type of error that can occur in an alignment (sequencing errors, indels, etc). In other words, in a spliced graph-alignment (*A*,*π*), we cannot have uninformative gap-factors, and the *δ* function assigns a cost 1 to each novel gap-factor and a cost 0 to all other factors and annotated gap-factors: thus ${\texttt {cost}}(A,\pi) = |\mathcal {G_{F}}|$ and Err(*A*,*π*)≤*β*. We focus on a bi-criteria version of the computational problem of computing the optimal *spliced graph-alignment* (*A*,*π*) of *R* to a graph $\mathcal {S}_{G}$, where first we minimize the cost, then we minimize the error. The intuition is that we want a spliced graph-alignment of a read that is consistent with the fewest novel splicing events that are not in the annotation. Moreover, among all such alignments we look for the alignment that has the smallest edit distance (which is likely due to sequencing errors and polymorphisms) in the non-empty regions that are aligned (i.e. the factors). Figure 5 shows an example of spliced graph-alignment of error value 2, and cost 2 — since it has two novel gap-factors.

In this paper we propose an algorithm that, given a read *R*, a splicing graph $\mathcal {S}_{G}$, and three constants, which are *L* (the minimum length of a MEM), *α* (the maximum alignment indel size), and *β* (the maximum number of allowed errors), computes an optimal spliced graph-alignment — that is, among all spliced graph-alignments with minimum cost, the alignment with minimum error. The next section details how ASGAL computes the optimal spliced graph-alignments of a RNA-Seq sample to the splicing graph $\mathcal {S}_{G}$, and how it exploits novel gap-factors to detect AS events.

### ASGAL approach

We now describe the algorithm employed by ASGAL to compute the optimal spliced graph-alignments of a sample of RNA-Seq reads to the splicing graph of a gene, to be used in order to provide the alternative splicing events supported by the sample and a measure of their quantification (i.e. the number of reads supporting the event).

**Fig. 6:**
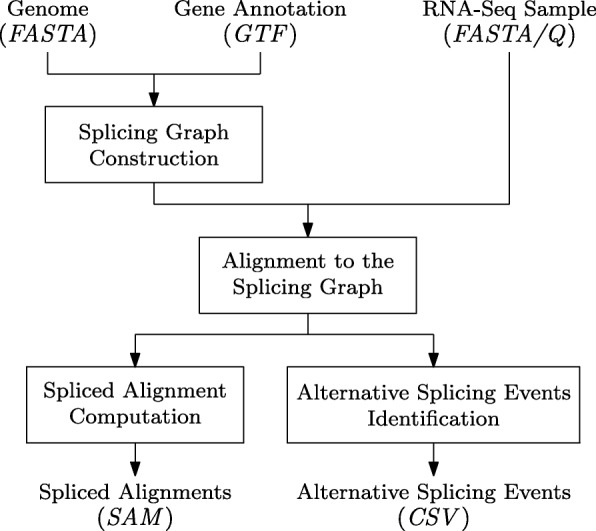
ASGAL pipeline. The steps of the pipeline implemented by ASGAL are shown together with their input and output: the splicing graph is built from the reference genome (FASTA file) and the gene annotation (GTF file), the RNA-Seq sample (FASTA or FASTQ file) is aligned to the splicing graph, and finally the alignments to the splicing graph are used to compute the spliced alignments to the reference genome (SAM file) and to detect the AS events supported by the sample (CSV file)

The ASGAL tool implements a pipeline consisting of the following steps: (1) construction of the splicing graph of the gene, (2) computation of the spliced graph-alignments of the RNA-Seq reads, (3) remapping of the alignments from the splicing graph to the genome, and (4) detection of the novel alternative splicing events. Figure 6 depicts the ASGAL pipeline.

In the first step, ASGAL builds the splicing graph $\mathcal S_{G}$ of the input gene using the reference genome and the gene annotation, and adds the novel edges to obtain the graph $\mathcal S^{\star }_{G}$ which will be used in the next steps.

The second step of ASGAL computes the spliced graph-alignments of each read *R* in the input RNA-Seq sample by combining MEMs into factors and gap-factors. For this purpose, we extend the approximate pattern matching algorithm of Beretta et al. [19] to obtain the spliced graph-alignments of the reads, which will be used in the following steps to detect novel alternative splicing events. As described before, we use the approach proposed by Ohlebusch et al. in [26] to compute, for each input read *R*, the set of MEMs between *Z*, the linearization of the splicing graph $\mathcal {S}_{G}$, and *R* with minimum length *L*, a user-defined parameter (we note that the approach of [26] allows to specify the minimum length of MEMs). We recall that the string *Z* is obtained by concatenating the strings seq(*v*) and *ϕ* for each vertex *v* of the splicing graph (recall that *ϕ* is the special character used to separate the vertex labels in the linearization *Z* of the splicing graph). We point out that the concatenation order does not affect the resulting alignment and that the splicing graph linearization is performed only once before aligning the input reads to the splicing graph.

Once the set *M* of MEMs between *R* and *Z* is computed, we build a weighted graph *G*_*M*_=(*M, E*_*M*_) based on the parameter *α*, representing the maximum alignment indel size allowed, and the two precedence relations between MEMs, ≺_*R*_ and ≺_*Z*_, respectively. Then we use such graph to extract the spliced graph-alignment. Intuitively, each node of this graph represents a perfect match between a portion of the input read and a portion of an annotated exon whereas each edge models the alignment error, the gap-factor of the spliced graph-alignment, or both. More precisely, there exists an edge from *m* to *m*^′^, with *m*,*m*^′^∈*M*, if and only if *m*≺_*R*_*m*^′^ and one of the following six conditions (depicted in Fig. 7) holds:

1. *m* and *m*^′^ are inside the same vertex label of *Z*, *m*≺_*Z*_*m*^′^, and either (i) *lgap*_*R*_>0 and *lgap*_*Z*_>0, or (ii) *lgap*_*R*_=0 and 0<*lgap*_*Z*_≤*α*. The weight of the edge (*m,m*^′^) is set to the edit distance between *sgap*_*R*_ and *sgap*_*Z*_ (Fig. 7a).

**Fig. 7:**
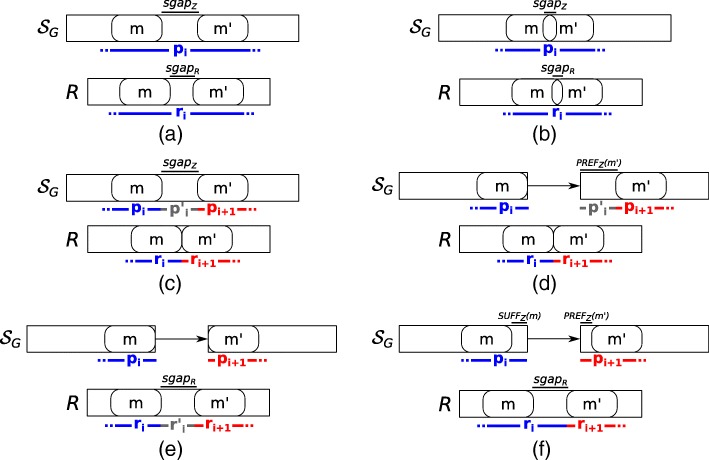
Conditions for linking two different MEMs. All the conditions used to connect two different MEMs and then to build the factors and gap-factors of a spliced graph-alignment are shown. In all the conditions, the first MEM must precede the second one on the read. In condition (**a**) and (**b**), the two MEMs occur inside the same vertex label and leave a gap (condition **a**) or overlap (condition **b**) on the read or on the vertex label. In these conditions, the two MEMs are joined in the same factor of the alignment. In condition **c**, instead, the two MEMs occur inside the same vertex label but they leave a long gap only on the vertex label and not on the read. In this case, the two MEMs belong to two different factors linked by a gap-factor. In the other conditions, instead, the two MEMs are inside the labels of two different vertices of the splicing graph, linked by a (possible novel) edge. For this reason, in any of these cases, the two MEMs belong to two different factors of the alignment. In condition **d**, the two MEMs leave a gap only the path, in condition **e** they leave a gap only on the read, and in condition **f**, they leave a gap on both the path and the read
2. *m* and *m*^′^ are inside the same vertex label of *Z*, *m*≺_*Z*_*m*^′^, *lgap*_*R*_≤0, and *lgap*_*Z*_≤0. The weight of the edge (*m,m*^′^) is set to |*lgap*_*R*_−*lgap*_*Z*_| (Fig. 7b).
3. *m* and *m*^′^ are inside the same vertex label of *Z*, *m*≺_*Z*_*m*^′^, *lgap*_*R*_≤0 and *lgap*_*Z*_>*α*. The weight of the edge (*m,m*^′^) is set to 0 (Fig. 7c).
4. *m* and *m*^′^ are on two different vertex labels seq(*v*_1_) and seq(*v*_2_), with *v*_1_≺*v*_2_, and *lgap*_*R*_≤0. The weight of the edge (*m*,*m*^′^) is set to 0 (Fig. 7d).
5. *m* and *m*^′^ are on two different vertex labels seq(*v*_1_) and seq(*v*_2_), with *v*_1_≺*v*_2_, *lgap*_*R*_>0, and SUFF_*Z*_(*m*)=PREF_*Z*_(*m*^′^)=*ε*. The weight of the edge (*m*,*m*^′^) is set to 0 if *lgap*_*R*_>*α*, and to *lgap*_*R*_ otherwise (Fig. 7e).
6. *m* and *m*^′^ are on two different vertex labels seq(*v*_1_) and seq(*v*_2_), with *v*_1_≺*v*_2_, *lgap*_*R*_>0, at least one between SUFF_*Z*_(*m*) and PREF_*Z*_(*m*^′^) is not *ε*. The weight of the edge (*m,m*^′^) is set to the edit distance between *sgap*_*R*_ and the concatenation of SUFF_*Z*_(*m*) and PREF_*Z*_(*m*^′^) (Fig. 7f).

Note that the aforementioned conditions do not cover all of the possible situations that can occur between two MEMs, but they represent those that are relevant for computing the spliced graph-alignments of the considered read. Intuitively, *m* and *m*^′^ contribute to the same factor (*p*_*i*_,*r*_*i*_) in cases 1 and 2 and the non-zero weight of the edge (*m,m*^′^) concurs to the spliced graph-alignment error. In cases 3-5, the edge (*m,m*^′^) models the presence of a novel gap-factor. More precisely, *m* contributes to the end of a factor (*p*_*i*_,*r*_*i*_) and *m*^′^ contributes to the start of the consecutive factor (*p*_*i*+1_,*r*_*i*+1_) and the novel gap-factor in between models an intron retention or a genomic deletion on an annotated exon (case 3), an alternative splice site shortening an annotated exon (case 4), and an alternative splice site extending an annotated exon or a new exon (case 5). Finally in case 6, *m* contributes to the end of a factor (*p*_*i*_,*r*_*i*_) and *m*^′^ contributes to the start of the consecutive factor (*p*_*i*+1_,*r*_*i*+1_) whereas the gap-factor in between can identify either a novel exon skipping event or an already annotated intron. In both these cases, the non-zero weight of the edge contributes to the spliced graph-alignment error.

The spliced graph-alignment of the read *R* is computed by a visit of the graph *G*_*M*_. More precisely, each path *π*_*M*_ of this graph represents a spliced graph-alignment and the weight of the path is the number of differences between the pair of strings in *R* and *Z* covered by *π*_*M*_. For this reason, for read *R*, we select the lightest path in *G*_*M*_, with weight less than *β* (the given error threshold) which also contains the minimum number of novel gap-factors, i.e. we select an optimal spliced graph-alignment.

The third step of ASGAL computes the spliced alignments of each input read with respect to the reference genome starting from the spliced graph-alignments computed in the previous step. Exploiting the annotation of the gene, we convert the coordinates of factors and gap-factors in the spliced graph-alignment to positions on the reference genome. In fact, observe that factors map to coding regions of the genome whereas gap-factors identify the skipped regions of the reference, i.e. the introns induced by the alignment, modeling the possible presence of AS events (see Fig. 3 for details). We note here that converting the coordinates of factors and gap-factors to positions on the reference genome is pretty trivial except when factors *p*_*i*_ and *p*_*i*+1_ are on two different vertices and only $p^{\prime }_{i}$ is *ε* (case *d-e* of Fig. 3). In this case, the portion $r^{\prime }_{i}$ must be aligned to the intron between the two exons whose labels contains *p*_*i*_ and *p*_*i*+1_ as a suffix and prefix, respectively. If $r^{\prime }_{i}$ aligns to a prefix or a suffix of this intron (taking into account possible errors within the total error bound *α*), then the left or right coordinate of the examined intron is modified according to the length of $r^{\prime }_{i}$ (Fig. 3d). In the other case (Fig. 3e), the portion $r^{\prime }_{i}$ is not aligned to the intron and it is represented as an insertion in the alignment. Moreover, the third step of our approach performs a further refinement of the splice sites of the introns in the obtained spliced alignment since it searches for the splice sites (in a maximum range of 3 bases with respect to the detected ones) determining the best intron pattern (firstly *GT-AG*, secondly *GC-AG* if *GT-AG* has not been found).

In the fourth step, ASGAL uses the set $\mathcal {I}$ of introns supported by the spliced alignments computed in the previous step, i.e. the set of introns associated to each gap-factor, to detect the novel alternative splicing events supported by the given RNA-Seq sample with respect to the given annotation. Let $\mathcal {I}_{n}$ be the subset of $\mathcal {I}$ composed of the introns which are not present in the annotation, that is, the *novel* introns. For each *novel* intron $\left [p_{s}, p_{e}\right ] \in \mathcal {I}_{n}$ which is supported by at least *ω* alignments, ASGAL identifies one of the following events, which can be considered one of the relevant events supported by the input sample:

- *exon skipping*, if there exists an annotated transcript containing two non-consecutive exons [ *a*_*i*_,*b*_*i*_] and [ *a*_*j*_,*b*_*j*_], such that *b*_*i*_=*p*_*s*_−1 and *a*_*j*_=*p*_*e*_+1.
- *intron retention*, if there exists an annotated transcript containing an exon [ *a*_*i*_,*b*_*i*_] such that (i) *a*_*i*_<*p*_*s*_<*p*_*e*_<*b*_*i*_, (ii) there exists an intron in $\mathcal {I}$ ending at *a*_*i*_−1 or *a*_*i*_ is the start of the transcript and (iii) there exists another intron in $\mathcal {I}$ starting at *b*_*i*_+1 or *b*_*i*_ is the end of the transcript.
- *alternative acceptor site*, if there exists an annotated transcript containing two consecutive exons [ *a*_*i*_,*p*_*s*_−1] and [ *a*_*j*_,*b*_*j*_] such that *p*_*e*_<*b*_*j*_, and there exists an intron in $\mathcal {I}$ starting at *b*_*j*_+1 or *b*_*j*_ is the end of the transcript.
- *alternative donor site*, if there exists an annotated transcript containing two consecutive exons [ *a*_*i*_,*b*_*i*_] and [ *p*_*e*_+1,*b*_*j*_] such that *p*_*s*_>*a*_*i*_, and there exists an intron in $\mathcal {I}$ ending at *a*_*i*_−1 or *a*_*i*_ is the start of the transcript.

**Fig. 8:**
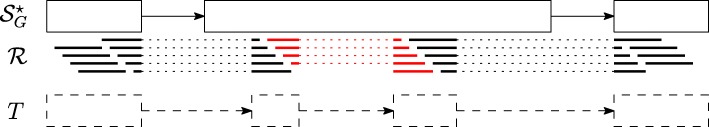
Example of false intron retention. The figure depicts a splicing graph $\mathcal {S}^{\star }_{G}$, a transcript *T*, and the alignments of a sample of reads from *T*. In this case, the transcript *T* shows a complex AS event w.r.t. the annotation of the splicing graph $\mathcal {S}_{G}^{\star }$ consisting of two new exons. ASGAL finds the new intron supported by the red alignments, but the analysis of the neighboring introns shows that no simple AS event can explain the alignments: this situation is recognized by ASGAL that refuses to make any prediction of a novel (surely incorrect) intron retention event

We note here that these definitions are accurately designed to minimize the chances of mistaking a complex AS event as those modeled with the notion of LSV for an AS event. For example, if we remove conditions (ii) and (iii) from the definition of intron retention, we could confuse the situation shown in Fig. 8 with an intron retention event.

### Genome-wide analysis

ASGAL is specifically designed to perform AS prediction based on a splice-aware alignment of an experiment of RNA-Seq reads against a splicing graph of a specific gene. The current version of ASGAL is time efficient when a limited set of genes are analyzed, while for genome-wide analysis we have implemented a pre-processing step that aims to speed up the process of filtering reads that map to genes under investigation. Given a set of genes and a RNA-Seq sample, this filtering procedure consists of three main steps: (i) the quasi-mapping algorithm of Salmon is first used to quantify the transcripts of the genes and to quickly assign each read to the transcripts, (ii) a smaller set of RNA-Seq samples, one for each gene, is then produced by analyzing the output of Salmon, and finally (iii) if the input sample contains paired-end reads, then the mate of the mapped reads that were not mapped by Salmon are then added to these smaller samples. Once we split the input RNA-Seq experiment in smaller samples, it is possible to use ASGAL on the different genes without having to align the entire sample of reads against each of them. We note that we decided to use Salmon as pre-processing step since it is very fast and it allows to split the input sample faster than any other spliced aligner. Anyway, in aligning reads to a reference transcriptome, some reads which cover unannotated exons are not aligned, excluding them from any downstream analysis: for this reason, future works will focus on improving this step.

## Results

In this section we will describe the experimental evaluation we performed to assess ASGAL ’s ability to align reads to a splicing graph and to detect alternative splicing event types. Such experimental analysis was performed on both simulated and real data. The former had the specific goal of measuring the quality of our tool in predicting specific AS event types, whereas the latter prove the ability of ASGAL to detect on real datasets annotated and novel alternative splicing events that are known to be RT-PCR validated.

We ran ASGAL using its default parameters in all the experiments. More precisely, the minimum length of the MEMs (*L*) was set to 15, while *α* and *β* were set to 3% of the maximum length of the input reads, and the minimum support for AS events (*ω*) was set to 3. The analyses were performed on a 64 bit Linux (Kernel 4.4.0) system equipped with Four 8-core IntelⓇ Xeon 2.30GHz processors and 256GB of RAM.

### Simulated data

In the first phase of our experimental analysis, we evaluated our tool using simulated data. The goal of this analysis was twofold: (i) to assess the accuracy and the efficiency of our method in aligning a RNA-Seq sample against a splicing graph, and (ii) to assess how well the method detects the alternative splicing events supported by a sample.

To avoid any bias in the experiments, we decided to reuse the same data — that is the reference genome, annotations, and RNA-Seq samples simulated with Flux [27] — used in [9]^1^. We considered two different RNA-Seq datasets of this corpus. More precisely, we downloaded two datasets: one composed of 5 million reads, and the other of 10 million reads. From now on, we will refer to these datasets as 5M and 10M, respectively. Each dataset covers 1000 randomly selected genes of the human GENCODE annotation (v19) [28]. We used AStalavista [6] (version 4.0) to extract the AS events included in the annotation of each gene, then we selected the genes whose annotation includes at least one AS event. After these filtering steps, the set of genes under analysis included 656 elements. Finally, we divided each read sample into 24 samples (by using the information included in the header of each entry of the file containing the reads), one for each chromosome, and we used cutadapt [29] (version 1.14) to remove poly-A tails.

**Fig. 9:**
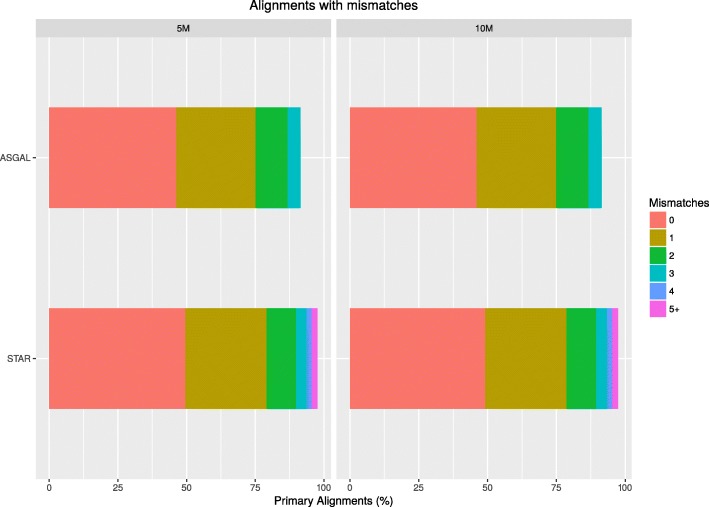
Mismatch frequencies. The figure shows for each considered sample (5M and 10M) and for each considered tool (ASGAL and STAR), the percentage of reads aligned divided by number of mismatches. The different colors indicates the number of mismatches

**Fig. 10:**
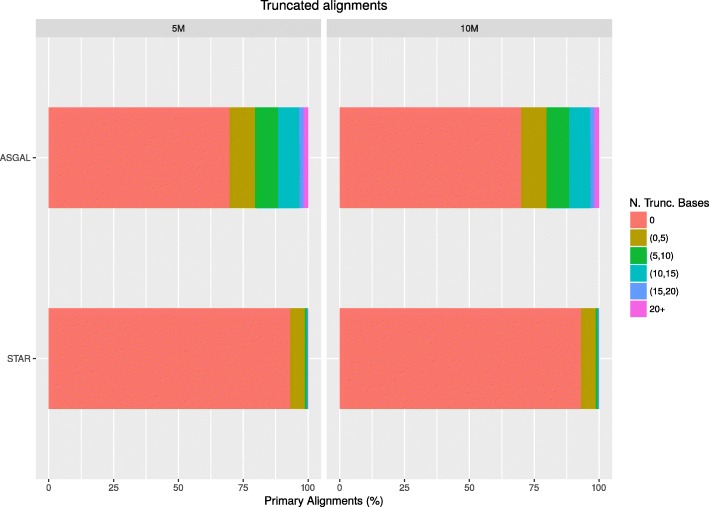
Reads truncation frequencies. The figure shows for each considered sample (5M and 10M) and for each considered tool (ASGAL and STAR), the percentage of incomplete alignments due to reads truncation. The different colors indicates the number of truncated bases

In the first part of our experimental analysis, we compared the alignment step of ASGAL with STAR [14] (version 2.5.4b), one of the best-known spliced aligner. Let us recall that ASGAL performs a splice-aware alignment and its current implementation is specifically designed to confirm or detect novel splice sites using the splicing graph as a main reference for the alignment of reads. Since our tool works at gene level — that is, it considers the splicing graph of each gene independently — we ran ASGAL on each gene independently whereas we ran STAR in two-pass mode on each chromosome, providing the annotation of the considered genes. We then selected all primary alignments reported by the two tools and we compared them using different metrics, as in [30]. Note that our tool considers each gene independently, thus it can align the same read to different genes and report multiple primary alignments of it, i.e. at most one for each gene. Table 1 reports the total number of mapped reads (91*%* for ASGAL, and 97*%* for STAR), as well as the number of alignments per read reported by the two tools. As expected, since we considered only primary alignments and since STAR aligns the input sample to the input reference, STAR yields a single alignment per read. Conversely, ASGAL might align the same read to different genes: in this case ASGAL outputs multiple primary alignments (at most one for each gene). However, this behaviour is extremely rare. Indeed, less than 0.2*%* of the considered reads are aligned to multiple genes. We also assessed the basewise accuracy of both ASGAL and STAR. As shown in Table 2, ∼98*%* of the primary alignments produced by both the tools map the read to the correct location, i.e. the read is placed in the position from where it were extracted. ASGAL produced fewer “Partially Mapped” alignments, i.e. the alignments which place some but not all the read bases in the correct positions, but more “Differently Mapped” alignments, i.e. the alignments which place all the read bases in positions different from those from which the read was simulated. Observe that the fewer “Partially Mapped” alignments is a consequence of the advantage of aligning directly to the splicing graph. Indeed, by investigating the “Partially Mapped” alignments of STAR, we found that the vast majority of these alignments (more than the 75*%*) place some read bases on an intron: this situation mainly occurs when the first (last) bases of an intron (exon) are equal to the first (last) bases of an exon (intron). By using the splicing graph, it is possible to avoid these situations since, when it is possible, it forces the alignments to be placed on the known exons of a gene. On the other hand, the higher number of alignments to positions different from the one of extraction is a consequence of the fact that ASGAL works at the gene level and can produce multiple primary alignments, of which only one aligns the read to the exact positions from which it was simulated. We also analyzed the number of incomplete alignments due to read truncation reported and each tool’s tolerance for mismatches. Figures 9 and 10 show the results of this analysis. As said before, ASGAL mapped 91*%* of the input reads whereas STAR mapped 97*%* of the reads. The differences in ASGAL alignments are due to two reasons: (i) with default parameters, the number of allowed errors in an alignment is smaller for ASGAL than for STAR (see Fig. 9), and (ii) ASGAL only maps reads to exonic regions, therefore it cannot correctly map reads covering intronic regions or long novel exons. Figure 10 reports the number of truncated alignments and we can note that ASGAL outputs more incomplete alignments than STAR. This is mainly due to the choice of parameter *L*. Indeed, ASGAL builds each alignment starting from anchors of at least *L* bases. If a prefix or a suffix of some read is not covered by any anchors, ASGAL outputs a truncated alignment since it cannot align that portion of the read. Although such behavior might remove parts of the alignments which are useful for the detection of alternative splicing events, we will show later that such aggressive truncation strategy does not affect significantly the following step of event identification. Moreover, at the cost of increasing the running time, it is possible to decrease the number of truncated alignments by setting a smaller value of *L*. Finally, we analyzed the computational resources required by the two tools. We ran both the tools using a single thread and we reported in Table 3 the total time and the memory peak required by them. As expected, since ASGAL works at the gene level, it required more time and less memory than STAR. Indeed, we ran STAR on each chromosome whereas we ran ASGAL on each gene and thus we needed to repeat its execution for the total number of processed genes. Moreover, we note that the slowest run of ASGAL on the 5M dataset took only 99 s whereas the slowest run on the 10M dataset took only 184 s. Since each run of ASGAL is independent from the others, by spreading a many-gene computation over multiple cores, we can reduce the running time, proportionally to the number of cores. We finally note that STAR can be run using multiple threads too. Nevertheless, we did not compare the time performance of ASGAL and STAR when run in parallel since our main goal is to measure the quality of the alignments.

**Table 1:** Number of alignments on the simulated datasets

Sample	Tool	Total reads	Unmapped	Number of alignments per read					Mapped reads
				1	2	3	4	5	
5M	ASGAL	3,226,895	281,767	2,938,383	6734	2	9	0	2,945,128
	STAR		75,947	3,150,948	0	0	0	0	3,150,948
10M	ASGAL	6,522,455	571,202	5,942,220	9009	13	10	1	5,951,253
	STAR		166,593	6,355,862	0	0	0	0	6355862

For each considered *Sample* (5M and 10M), the number of reads simulated from the 656 considered genes is shown along with the number of *Unmapped* reads, the number of reads mapped only once, the number of reads mapped multiple times, and the total number of *Mapped Reads* by the considered *Tool* (ASGAL and STAR)

**Table 2:** Read placement accuracy on the simulated datasets

Sample	Tool	Alignments	Perfectly mapped	Partially mapped	Differently mapped
5M	ASGAL	2,951,893	2,907,881 (98.51*%*)	34,047 (1.15*%*)	9965 (0.34*%*)
	STAR	3,150,948	3,072,183 (97.50*%*)	76,599 (2.43*%*)	2166 (0.07*%*)
10M	ASGAL	5,960,322	5,879,604 (98.65*%*)	66,463 (1.11*%*)	14,255 (0.24*%*)
	STAR	6,355,862	6,201,270 (97.56*%*)	150,373 (2.37*%*)	4219 (0.07*%*)

For each considered *Sample* (5M and 10M) and for each considered *Tool* (ASGAL and STAR), the number of *Alignments* produced by each tool is shown along with the number of *Perfectly Mapped* alignments, i.e. the alignments which place all the read bases in the correct position, the number of *Partially Mapped* alignments, i.e. the alignments which place some (but not all) read bases in the correct position, and the number of *Differently Mapped* alignments, i.e. the alignments which place all the read bases in a position different from the one from which the read has been simulated

**Table 3:** Computational resources required by the two tested methods (ASGAL and STAR) to align the simulated datasets (5M and 10M)

Sample	Tool	Time (m)	Memory Peak (MB)
5M	ASGAL	475	249
	STAR	179	7921
10M	ASGAL	945	247
	STAR	197	7921

These results are shown in terms of time (minutes) and memory peak (MegaBytes). These results take into account both the index and the alignment steps performed by the tools

In the second part of our experimental analysis on simulated data, we compared the event identification step of ASGAL with three other well-known tools for the detection of AS events from RNA-Seq data: SplAdder [9] (version 1.0.0), rMATS [12] (version 4.0.2 turbo), and SUPPA2 [11] (version 2.3). Note that we did not include MAJIQ [13] in the experimental comparison since the tool focuses on Local Splicing Variations (LSV). Although LSVs capture previously defined types of alternative splicing as well as more complex transcript variations, MAJIQ does not provide a direct way to map one kind of event into the other one. Moreover, we did not include LeafCutter [16] in our experiments since the tool focuses on introns which model complex splicing events and there is no easy way to extract from them the simpler AS events.

**Fig. 11:**
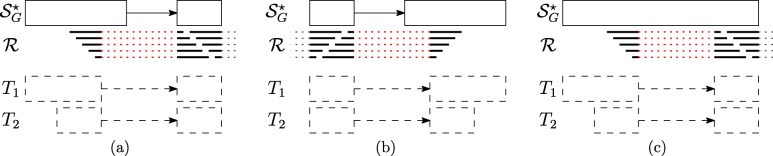
Examples of splicing event misclassification. The figure depicts three situations in which ASGAL may detect a false positive event: an alternative donor site in (**a**), an alternative acceptor site in (**b**), and an intron retention in (**c**). The black arrows represent an annotated intron (in the first two cases), whereas the red dotted lines represent the novel intron supported by the input sample with respect to the annotation $\mathcal {S}_{G}^{\star }$. In cases (**a**) and (**b**), the novel event induces an alternative 5’ and an alternative 3’ splice site (respectively) with respect to the intron in the annotation, and in case (**c**) the novel intron is inside an already annotated exon. On the assumption that the reads come from a hypothetical novel transcript *T*_1_, then ASGAL outputs a true positive event. Indeed, all the events refer to an annotated exon, thus one (the start of the 5’ exon in case (**a**) and the end of the 3’ exon in case (**b**)) or two splice sites (the start of the 5’ exon and the end of the 3’ exon in case (**c**)) involved in the predicted event are already annotated in $\mathcal {S}_{G}^{\star }$. By contrast, if the aligned reads come from a hypothetical novel transcript *T*_2_, then ASGAL produces a false positive event: it outputs the events with respect to the annotated exon but the true events refer to a novel exon having both splice sites not annotated in $\mathcal {S}^{\star }_{G}$

The main goal of our experimental analysis on simulated data was to evaluate the ability of our tool in detecting splicing event types that are novel with respect to the input annotation, i.e. splicing events that are not already contained in the input annotation. For this reason, in the first part of our analysis, we created a set of reduced annotations by removing some transcripts from the annotations of the considered genes. In such a way, by providing the tools with these reduced annotations and a read sample containing reads simulated from the original annotation, we assessed how well they are able to detect novel event types, i.e. events that come from transcripts not contained in the reduced annotation but that are supported by the input RNA-Seq data. Observe that ASGAL is specifically designed to enrich a gene annotation with novel events supported by a RNA-Seq sample and thus this first analysis better reflects the performance of ASGAL. On the other hand, we wanted to perform a comparison also with current state of art tools that specifically detects from differential analysis of RNA-Seq experiments AS events that are already present in a gene annotation or use annotated splice sites, such as SUPPA2 and rMATS. For this purpose, we set up a second analysis in which we used ASGAL to detect from the read alignments introns that support annotated splicing events that may be alternative to a given isoform. More precisely, we used ASGAL to confirm the presence of annotated introns in the RNA-Seq experiment that induce exon skipping, or alternative splice sites or intron retention events that are already in the splicing graph. For this purpose, we provided the tools with the original annotations of each considered gene and the same read samples used in the previous analysis. As said above, ASGAL is designed to detect events that are novel with respect to the input gene annotation. For this reason, even in the annotated case, ASGAL looks for potential novel AS events by extracting from the alignments to the splicing graph those introns that may support the presence in the experiment of an isoform related to an event that is alternative with respect to an already annotated isoform. For example, an alternative splicing event may be reported by ASGAL if it is supported by an annotated intron that belongs to an isoform whose splice sites are alternative to another annotated isoform. However, by using only the computed set of introns to detect AS events, ASGAL ’s events prediction may show a higher number of false positives, as illustrated in Fig. 11: even though the computed alignments are correct, some introns can be misclassified as alternative splice site or intron retention. More in detail, this misclassification occurs when the considered intron is the first (or the last) intron of a transcript: in these cases, by using only the information provided by the introns, it is not possible to fully understand if the considered intron supports a real AS event. A planned improvement of ASGAL is to refine the procedure for identifying and classifying AS events by analyzing the coverage: this would allow to correctly classify the events of Fig. 11.

As anticipated, one of the tools we compared with is SplAdder, a software for identifying and quantifying alternative splicing events starting from a given annotation and the alignment files [9]. Although SplAdder might look similar to ASGAL, it performs different tasks. More precisely, as confirmed by our experiments, SplAdder builds a splicing graph starting from a given annotation and enriches it by exploiting the spliced alignments, but to identify the AS events it requires that all the isoforms involved in the event are supported by reads in the experiment. On the other hand, ASGAL is able to identify the alternative splicing events even if only a single isoform inducing the event is expressed in the experiment, since it uses the annotation as a reference for the identification of the novel AS events. This case is especially important, since usually there is a single transcript expressed per gene, when considering only a sample [31]. According to SplAdder ’s supplementary material, the default behaviour of SplAdder can be modified by adapting different parameters that guide the confirmation process of each alternative splicing event found. However, it is not an easy task to modify these parameters since they are hard-coded and it is not even clear how to choose the best values without the risk of introducing undesired behaviors. The other tools involved in the comparison, that are rMATS and SUPPA2, aim to detect the differential splicing events between conditions. More precisely, rMATS implements a statistical method for the detection of differential alternative splicing from replicate RNA-Seq data. A statistical test can also be performed to assess whether a difference in the isoform ratio of a gene between two conditions is significant with respect to a given threshold, also taking into account biological replicates. rMATS starts from either a RNA-Seq dataset or an alignment file produced by a spliced aligner, and produces as output a file for each considered AS event, obtained from the annotation and the samples. Similarly, SUPPA2 is able to infer the differential AS events from RNA-Seq data across multiple conditions and taking into account their biological variability. SUPPA2 starts from the abundances of each transcript in the considered annotation, expressed in transcript per million (TPM) units, and quantifies the AS events in terms of proportion spliced in (psi) for each sample. The differential splicing is given in terms of the differences of these relative abundances for each condition. As stated in [11], the quantification of the abundances values are computed by using Salmon [15] (in our analysis, we used version 0.9.1).

As done in [9], to assess the accuracy of the tools in detecting novel alternative splicing event types, we provided a reduced annotation, obtained in the following way. First of all, we used AStalavista [6] to extract all the alternative splicing events contained in the annotations of the 656 considered genes. This resulted in a total of 2568 alternative splicing events: 1574 exon skippings, 416 alternative acceptor sites, 290 alternative donor sites, and 288 intron retentions. Then, for each gene and for each event identified by AStalavista, we created a new reduced annotation containing all the transcripts except those responsible for such event. Here, we focused our attention on exon skippings, alternative splice sites (both acceptor and donor), and intron retentions caused by the insertion of a new intron inside an exon. Since the alternative splice site can consist of both shortening or extending an exon, we added both these cases to the events considered in the experimental evaluation. On the other hand, we did not consider the possible insertion of a new exon inside an intron and the intron retention caused by the union of two exons, since detecting such events is impossible using only data from the splicing graph. Moreover, when different events on the same gene produced the same reduced annotation, we considered the annotation only once. We obtained a total of 3274 AS events and 2792 reduced annotations. Differently from [9], where the reduced annotation provided as input to the tools contained only the first transcript of the annotation of each gene, we generated the gene annotations by keeping all the transcripts except the ones including the intron supporting the considered AS event. Indeed, ASGAL is less general than SplAdder in detecting novel exons that appear in intronic regions and that can only be detected by aligning reads to large intronic region. The exons involved in the considered novel events must be present in the reduced annotation to allow ASGAL to detect them. However, observe that ASGAL uses the genomic regions close to exon splice sites to detect novel exons that are variants of existing ones.

For each gene and for each reduced annotation, we ran all tools on the two considered datasets of reads, namely 5M and 10M. We recall here that these RNA-Seq samples were simulated with Flux simulator, cover 1000 randomly selected human genes and were used in the experimental analysis performed in [9]. For each type of alternative splicing event we analyzed the predictions over the set of 656 genes, computing the corresponding values of precision, recall, and F-measure. More precisely, given a gene and its reduced annotation, we consider as ground truth the set of events found by AStalavista in the original annotation. To compute the values of precision, recall, and F-measure, we considered the number of events that are in the original annotation but not in the reduced one and are found by the tools as true positives, the number of events that are in the original annotation but not in the reduced one and are *not* found by the tool as false negatives, and the number of events found by the tools and not in the annotation as false positives. We reported in Table 4 the quality results, for the different alternative splicing events, obtained by ASGAL, SplAdder and rMATS (for which we used STAR to compute the alignments), and SUPPA2 (for which we used Salmon to obtain the transcript quantification). The results show that ASGAL achieved the best values of precision, recall and F-measure in almost all the alternative splicing event types with the only exception of the recall of the alternative splice sites (A5 and A3). We investigated those cases and we found that our method applies strict criteria in detecting the alternative splice site events that extend an annotated event. As previously described, to detect this kind of event, ASGAL requires that the reads align leaving a gap on them, and requires the presence of sufficiently long anchors on two different exons, typically 15bps related to the length *L*. As a consequence, our method detects an alternative splice site event extending an exon when the length of the extension does not exceed the length of the read minus twice the length of *L* (the two anchors). By these requirements our method may not be able to detect alternative splice site extending an exon of several bases, as observed in the cases analyzed in our experimental analysis: for this reason ASGAL shows a lower recall on alternative splice site events. However, our method achieved the best values of F-Measure in all the alternative splicing event types — hinting that our criteria are well balanced — highlighting the ability of ASGAL in detecting novel alternative splicing events. To better analyze our results, we decided to manually examine the exon skipping events found by ASGAL but not by the other tools. In this analysis, we considered only SplAdder and rMATS since SUPPA2 did not find any novel exon skipping event. We found that most of the events not detected by SplAdder are due to the fact that only one of the two isoforms involved in the event is supported by the input alignments. As said above, to confirm an AS event, SplAdder requires that all the isoforms involved in the event are supported by reads in the experiment. Regarding rMATS, instead, we found that it does not output most of the events involving the skip of multiple exons. Moreover, by increasing the number of reads in the input set, all methods almost always achieve better recall with a slightly worse precision, since a higher coverage allows to detect a higher number of supported introns that are used to detect AS events. As it is possible to notice from Table 4, results of both rMATS and SUPPA2 are zero: this was expected since these two methods are not designed to detect novel events. More precisely, rMATS is not able to detect AS events involving novel splice sites (for this reason, it is able to detect only exon skipping events) whereas SUPPA2 only detects AS events that are present in the input annotation. To this purpose, as anticipated before, in order to have a thorough comparison with these tools, we also ran an experimental analysis considering all the AS events present in the annotation. More precisely, we ran all the tools providing them the full annotation of the genes. Table 5 reports the results obtained in this setting. Results of the tools are similar, with ASGAL performing slightly better on exon skipping events, while SUPPA2 outputs better predictions of alternative donor and acceptor sites, and both SUPPA2 and rMATS have better results on intron retention events. By a careful inspection of the results obtained by ASGAL, we observed that more than 85% of the false positives alternative splice site events in Table 5 and more than 98% of false positives intron retention events are due to the cases discussed previously and illustrated in Fig. 11, i.e. the events are induced by the first or the last intron of a transcript. Consequently, a lower precision cannot be imputed to the quality of the alignment performed by ASGAL. Though the false positive cases described above could be eliminated by using more conservative rules: the actual rules used by ASGAL produce higher precision in detecting novel event types as shown in Table 4 as well as good results for RT-PCR validated AS events discussed in the next section.

**Table 4:** Quality measures in detecting novel alternative splicing events on the simulated datasets with 5M and 10M reads

		5M				10M			
Tool	Measure	ES	A3	A5	IR	ES	A3	A5	IR
ASGAL	Prec	0.997	0.955	0.905	0.862	0.995	0.938	0.895	0852
	Rec	0.917	0.741	0.737	0.674	0.963	0.789	0.781	0.681
	FM	0.955	0.835	0.812	0.756	0.979	0.857	0.834	0.757
SplAdder	Prec	0.885	0.612	0.475	0.299	0.874	0.642	0.495	0.272
	Rec	0.802	0.884	0.821	0.531	0.848	0.925	0.891	0.521
	FM	0.841	0.723	0.602	0.383	0.860	0.758	0.637	0.357
rMATS	Prec	0.996	-	-	-	0.997	-	-	-
	Rec	0.860	-	-	-	0.863	-	-	-
	FM	0.923	-	-	-	0.925	-	-	-
SUPPA2	Prec	-	-	-	-	-	-	-	-
	Rec	-	-	-	-	-	-	-	-
	FM	-	-	-	-	-	-	-	-

Results obtained by ASGAL, SplAdder and rMATS (for which we used STAR to compute the alignments), and SUPPA2 (for which we used Salmon to obtain the transcript quantification) are reported. Precision (Prec), Recall (Rec), and F-Measure (FM) achieved on the simulated datasets in detecting novel alternative splicing events: exon skipping (ES), alternative acceptor site (A3), alternative donor site (A5), and intron retention (IR). A dash “-” means that the considered tool is not designed to detect that type of *novel* AS events

**Table 5:** Quality measures in detecting annotated alternative splicing events on the simulated datasets with 5M and 10M reads

		5M				10M			
Tool	Measure	ES	A3	A5	IR	ES	A3	A5	IR
ASGAL	Prec	0.999	0.850	0.702	0.657	0.999	0.846	0.703	0.642
	Rec	0.924	0.788	0.774	0.719	0.966	0.814	0.799	0.722
	FM	0.960	0.818	0.736	0.687	0.982	0.830	0.748	0.680
SplAdder	Prec	0.963	0.844	0.734	0.513	0.957	0.857	0.733	0.450
	Rec	0.822	0.927	0.899	0.552	0.855	0.947	0.936	0.531
	FM	0.887	0.884	0.808	0.532	0.903	0.900	0.822	0.487
rMATS	Prec	0.995	1	1	0.976	0.996	1	1	0.976
	Rec	0.905	0.685	0.755	0.830	0.905	0.685	0.755	0.830
	FM	0.948	0.813	0.860	0.897	0.949	0.813	0.860	0.897
SUPPA2	Prec	1	0.880	0.754	0.976	1	0.880	0.754	0.976
	Rec	0.894	1	1	0.830	0.894	1	1	0.830
	FM	0.944	0.936	0.860	0.897	0.944	0.936	0.860	0.897

Results obtained by ASGAL, SplAdder and rMATS (for which we used STAR to compute the alignments), and SUPPA2 (for which we used Salmon to obtain the transcript quantification) are reported. Precision (Prec), Recall (Rec), and F-Measure (FM) achieved on the simulated datasets in detecting annotated alternative splicing events: exon skipping (ES), alternative acceptor site (A3), alternative donor site (A5), and intron retention (IR)

Finally, we discuss the efficiency of the tested methods. To this purpose, we retrieved the running time and the maximum memory used by each tool in the detection of annotated events on the 10M dataset using the GNU time command. We decided to analyze the performance during the detection of annotated events to have a fair comparison, since rMATS and SUPPA2 are meant to detect only annotated events and do not provide any useful information when detecting novel events. As described before, ASGAL is composed of two main steps: the alignment of the reads and the detection of the events. These two steps required 945 and 60 min, respectively, and the main memory usage was 760 MB. For SplAdder we considered the time required by STAR to align the reads to the chromosomes and by the tool itself to detect the events. The first step required 45 min whereas the second step required 36 min. The main memory usage was 5.8 GB and was due to the alignment step. Similarly to SplAdder, rMATS aligns the reads using STAR. Therefore the first step of rMATS requires the same time as SplAdder (45 min). Detecting the events using rMATS, on the other hand, requires 268 min. The main memory usage of this tool was, again, due to the alignment step of STAR and was equal to 5.8 GB. Finally, SUPPA2 is composed of three main steps: quantifying the transcripts using Salmon, generating events from the annotation using SUPPA2generate, and computing the psi-value, i.e. the relative abundance value per sample, of the events using SUPPA2psi. These steps required 130, 19, and 19 min respectively. The main memory usage was due to the quantification step of Salmon and was equal to 192 MB. Note that in the previous analysis we did not consider the time required by STAR and Salmon to index the genome and the transcriptome, respectively. In fact, such indexes could already be available from previous runs of the tools. Nevertheless, we report the times for these steps for sake of completeness. STAR required 152 min and 7.9 GB to index the chromosomes of the Human Genome whereas Salmon required 2 min and 14 MB to index its transcriptome.

### Real data

We also applied our method to a real dataset of RNA-Seq reads in order to assess its performance in detecting events from RNA-Seq data. Inspired by the experimental analysis performed in the SUPPA2 paper [11], we considered a set of 83 RT-PCR validated AS events upon TRA2A and TRA2B knockdown compared to control sets from the study in [32]. More precisely, this experiment consists of 3 samples^2^ in which there is a double knockdown of the TRA2A and TRA2B splicing regulatory proteins and 3 control datasets^3^. The goal of the experimental analysis of SUPPA2 in [11] was to identify the 83 RT-PCR validated AS events in these knockdown versus control datasets. Since in 2 of these 83 events the positions of the intron(s) involved in the event were missing, they could not be used to compare the predictions of the tools. For this reason, we decided to remove such 2 events from the set, resulting in a set of 81 events on which we tested ASGAL, rMATS, SUPPA2 and SplAdder, with the specific goal of identifying the RT-PCR validated alternative splicing events. More precisely, we ran all tools on the 3 replicate datasets with the knockdown of the two splicing regulatory proteins (SRR1513332, SRR1513333, and SRR1513334).

**Fig. 12:**
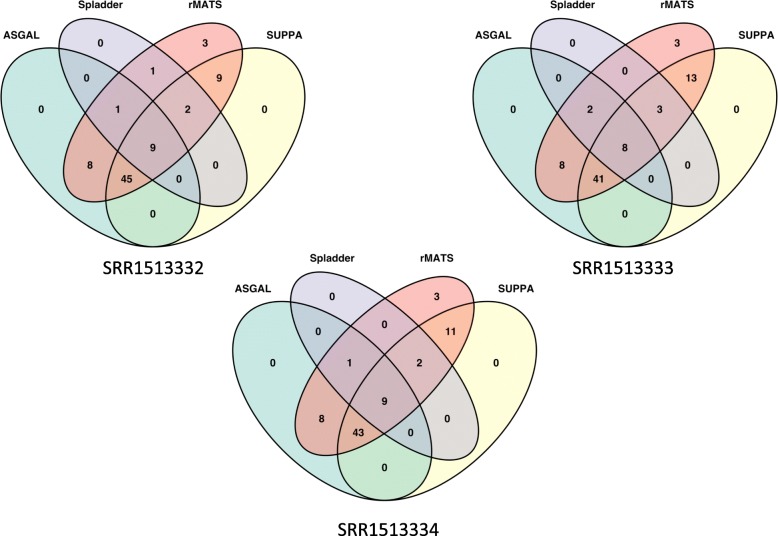
Results on RT-PCR validated events. Venn diagram showing the overlaps in results obtained by ASGAL, SUPPA2, rMATS, and SplAdder, on the 3 knockdown dataset. The results are expressed as the number of RT-PCR validated events detected by the various tools

**Fig. 13:**
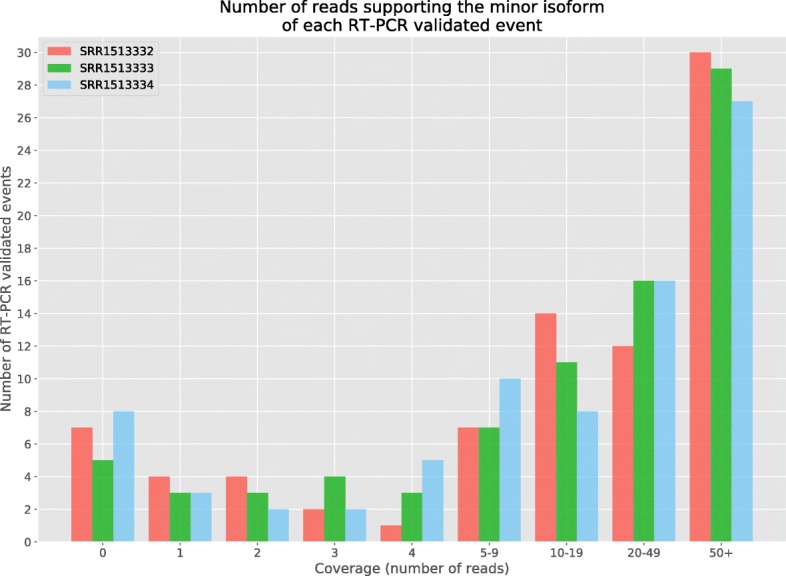
Coverage of RT-PCR validated events. Bar chart showing the coverage of the minor isoform of each RT-PCR validated events. The coverage is expressed as the number of spliced alignments supporting the intron that skips the exon(s)

We ran ASGAL in “genome-wide” mode on each dataset, to analyze reads that are potentially from the entire genome. Moreover, we ran SUPPA2 based on the quantifications obtained with Salmon, while we provided the alignments obtained with STAR to SplAdder and rMATS. We compared the results obtained on each dataset with the tested methods and, in particular, we considered all the events output by such tools that were in the list of events under analysis. In Fig. 12 we show a comparison of the results obtained by the tools on the 3 knockdown datasets. As it is possible to observe, rMATS was the tool that was able to detect more events (78 on SRR1513332, 78 on SRR1513333, and 77 on SRR1513334). Similarly, SUPPA2 identified 65 events in each of the 3 datasets, while ASGAL predicted 63,59, and 61 events on the SRR1513332, SRR1513333, and SRR1513334 dataset, respectively. Finally, SplAdder was able to identify only 13, 13, and 12 RT-PCR validated AS events on the SRR1513332, SRR1513333, and SRR1513334 dataset, respectively. We note that the events not identified by ASGAL show an extremely low support. To better validate the results obtained by ASGAL, we extracted from the alignments computed with STAR the spliced alignments supporting each considered event. More in detail, since each RT-PCR validated event is an exon skipping event, we counted the number of spliced alignments supporting the exclusion isoform of each event, i.e. the spliced alignments supporting the intron that confirms the skipping of one or more exons. We report the result of this analysis in Fig. 13, where the coverage of the 81 considered RT-PCR validated events is shown. These results highlight the ability of ASGAL in predicting AS events from real datasets of RNA-Seq reads, especially if compared to SplAdder which is the most similar tool. We note one more time that, to detect an AS event, SplAdder needs that all the isoforms involved in the event are supported by the reads in the sample. Thus, we analyzed how many of the RT-PCR validated events are supported by a single isoform. The obtained results show that, in the three considered datasets, 59, 54, and 65 events, respectively, are not supported by both the isoforms involved in the event. Moreover, the performances achieved by ASGAL on the tested datasets are similar, in terms of the number of detected AS events, to that of SUPPA2, which still lacks in detecting the novel events. The only tool that slightly outperformed ASGAL in this experimental analysis is rMATS. Anyway, also this latter method is not able to detect AS events involving novel splice sites that are not already in the considered annotation. Moreover, ASGAL identified more AS events than the RT-PCR validated ones. We analyzed the genes involved in the 81 considered RT-PCR validated AS events and we checked if the events additionally reported by ASGAL were also detected by the other considered tools. Table 6 summarizes the results of this analysis: except for intron retention events, the majority of the AS events identified by ASGAL were also reported by rMATS and SUPPA2.

**Table 6:** For each considered knockdown datasets and for each considered event type, we reported the number of AS events identified by ASGAL with respect to the considered genes, i.e. the genes involved in the 81 considered RT-PCR validated AS events, and how many of these events were also identified by the other tools considered in our analysis

	SRR1513332				SRR1513333				SRR1513334			
	ES	A3	A5	IR	ES	A3	A5	IR	ES	A3	A5	IR
ASGAL	343	168	141	56	356	170	138	50	323	149	140	51
SplAdder	33	18	3	2	35	17	2	1	28	18	2	1
rMATS	314	65	48	18	328	65	54	16	302	63	46	18
SUPPA2	193	83	78	17	191	82	78	16	189	83	76	17

Finally, we ran our tool providing a reduced annotation. As done in our experiments on simulated data, we removed all the transcripts which include the introns supporting the considered event and we used this annotation as input for our tool. In this way, we could test the ability of our tool in detecting novel AS events, not already present in the input annotation. In this setup, ASGAL predicted the same events predicted using the full annotation, i.e. 63, 59, and 61 events on the SRR1513332, SRR1513333, and SRR1513334 dataset, respectively.

Overall, ASGAL proved to be a competitive tool to detect annotated AS events, while allowing also the possibility of predicting the novel ones.

## Conclusions

In this paper we proposed ASGAL, a tool for predicting alternative splicing (AS) events from a RNA-Seq sample and a gene annotation given by a collection of annotated transcripts. ASGAL differs from similar tools since it implements a splice-aware algorithm for mapping RNA-Seq data to a splicing graph. The alignments of the reads to the splicing graph are then analyzed to detect differences, at the intron level, between the known annotation and the introns obtained by the alignments, in order to reconstruct AS events. Indeed, tools for AS prediction rely on a previously computed spliced-alignment of reads to a linear reference genome. While the spliced-alignment to a reference is a well understood notion, in this paper we investigated the problem of optimally mapping reads to a splicing graph by formalizing the notion of *spliced graph-alignment*. Then we proposed an algorithmic approach to compute optimal spliced graph-alignments. Indeed, the graph aligner module of ASGAL can be used independently to produce spliced graph-alignments of RNA-Seq reads to a general splicing graph. Note that our notion of spliced graph-alignment is tailored for detecting AS event types that are either simple or a combination of two different simple events. However, such a notion deserves to be further investigated to detect more complex combinations of AS event types. This will be the goal of a future development of the tool. Future works will also focus on an in-depth analysis of the influence of the parameter *L* on the overall accuracy and efficiency of our tool in the alignment step, as well as in predicting events. Another further step will focus on allowing ASGAL to be used natively in a genome-wide analysis by improving its pre-processing step or by directly improving its code.

To the best of our knowledge, ASGAL is the first tool for computing the splice-aware alignment of RNA-Seq data to a splicing graph. Compared with current tools for the spliced alignment to a reference genome, ASGAL produces high quality alignments to the splicing graph, even though there is still room for further improvements, for example in the direction of using ASGAL for confirming AS events already contained in the input annotation and for predicting novel exons that may be detected by insertions in the alignment of reads to the splicing graph that match to intronic regions of the reference genome.

The experimental analysis discussed in this paper shows some advantages of ASGAL in using a splice-aware aligner of RNA-Seq data to detect alternative splicing events that are novel with respect to the annotation of a splicing graph, i.e. the events which involve either novel or annotated splice sites. In this sense, ASGAL can be used to enrich a given annotation with novel alternative splicing events in order to allow a downstream tool for differential alternative splicing analysis such as SUPPA2 to also quantify these new events.

Compared to other approaches, ASGAL can work in presence of a poor gene annotation given by the splicing graph to enrich its structure with novel events. A natural extension of the ASGAL alignment method is the detection of AS events in a de-novo framework, where only the reference transcriptome is known. A future direction that we will investigate is the extension of ASGAL to work on a FASTA file input containing only mRNA sequences related to a reference transcriptome. In this case, a draft splicing graph may be built from the FASTA file and then used to infer AS events by using the ASGAL procedure.

A problem which is related to mapping RNA-Seq reads to a splicing graph is mapping genomic reads directly to a graph representation of multiple genomes (pan-genome): this problem is tackled by vg [33]. Despite this, how to apply a read mapper to a pan-genome graph for transcriptome analysis remains an interesting open problem.

## Abbreviations

A3: Alternative acceptor splice site
A5: Alternative donor splice site
AS: Alternative splicing
ASGAL: Alternative splicing graph ALigner
bps: Base pairs
ES: Exon skipping
EST: Expressed sequence tag
CSV: Comma-separated values
FM: F-Measure
GB: GigaByte
GTF: Gene transfer format
IR: Intron retention
LSV: Local splicing variations
MB: MegaByte
MEM: Maximal exact match
Prec: Precision
PSI: Proportion spliced in
Rec: Recall
RNA-Seq: RNA sequencing
RT-PCR: Reverse transcriptase polymerase chain reaction
SAM: Sequence alignment map
TRA2A: Transformer-2 protein homolog Alpha
TRA2B: Transformer-2 protein homolog Beta

## Footnotes

1 Data available at http://public.bmi.inf.ethz.ch/projects/2015/spladder/

2 SRA accession numbers SRR1513332, SRR1513333, and SRR1513334.

3 SRA accession numbers SRR1513329, SRR1513330, and SRR1513331.
